# Supplementary material for: Susceptibility of Human Oral Squamous Cell Carcinoma (OSCC) H103 and H376 cell lines to Retroviral OSKM mediated reprogramming
Source: PeerJ. 2017 Apr 13;5:e3174. doi: 10.7717/peerj.3174 (PMC5392249; doi:10.7717/peerj.3174)
Supplement: Data S1 — Quantitative data obtained by RT-qPCR from H103 RNA [file peerj-05-3174-s001.doc]

**REPLICATE** 1

|  | **Gene** | **Average C_T_ (target gene)** | **Average C_T_ t (ACTB)** | **∆ C_T_ = Target Gene - ACTB** | **∆∆ C_T_ = ∆ C_T_ Target Gene - ∆Ct Control** | **2^-∆∆^ ^CT^** |
| --- | --- | --- | --- | --- | --- | --- |
| **Parental H103** | **Oct4** | 34.08 ± 0.68 | 18.95 ± 0.44 | 15.13 ± 0.81 | 0 ± 0.81 | 1 |
|  | **Sox2** | 33.93 ± 1.50 | 18.95 ± 0.44 | 14.98 ± 0.44 | 0 ± 0.44 | 1 |
|  | **Klf4** | 27.83 ± 0.85 | 18.95 ± 0.44 | 8.88 ± 0.96 | 0 ± 0.96 | 1 |
|  | **C-Myc** | 24.99 ± 0.84 | 18.95 ± 0.44 | 6.04 ± 0.95 | 0 ± 0.95 | 1 |
|  | **Nanog** | 36.69 ± 0.21 | 18.95 ± 0.44 | 17.74 ± 0.49 | 0 ± 0.49 | 1 |
| **IPSC**  **H103**  **(P5)** | **Oct4** | 34.50 ± 0.65 | 18.54 ± 0.56 | 15.96 ± 0.86 | 0.83 ± 0.86 | 0.81 |
|  | **Sox2** | 27.63 ± 0.68 | 18.54 ± 0.56 | 9.09 ± 0.88 | -5.89 ± 0.88 | 59.30 |
|  | **Klf4** | 30.92 ± 0.43 | 18.54 ± 0.56 | 12.38 ± 0.86 | 3.50 ± 0.86 | 0.09 |
|  | **C-Myc** | 32.07 ± 0.43 | 18.54 ± 0.56 | 13.53 ± 0.86 | 7.49 ± 0.86 | 0.01 |
|  | **Nanog** | 34.74 ± 0.47 | 18.54 ± 0.56 | 16.20 ± 0.73 | -1.54 ± 0.73 | 2.91 |
| **IPSC**  **H103 (P10)** | **Oct4** | 32.75 ± 0.35 | 18.89 ± 0.29 | 13.86 ± 0.45 | -1.27 ± 0.45 | 2.41 |
|  | **Sox2** | 27.40 ± 1.52 | 18.89 ± 0.29 | 8.51 ± 1.55 | -6.47 ± 1.55 | 88.65 |
|  | **Klf4** | 30.80 ± 0.44 | 18.89 ± 0.29 | 11.91 ± 0.53 | 3.03 ± 0.53 | 0.12 |
|  | **C-Myc** | 31.25 ± 0.50 | 18.89 ± 0.29 | 12.36 ± 0.58 | 6.32 ± 0.58 | 0.01 |
|  | **Nanog** | 33.76 ± 1.83 | 18.89 ± 0.29 | 14.87 ± 1.85 | -2.87 ± 1.85 | 7.31 |

**Fold Change of Pluripotent Genes Expression in Reprogrammed H103 Relative to Parental by ∆∆Ct Method**

Sample

Sample

**REPLICATE** 2

|  | **Gene** | **Average C_T_ (target gene)** | **Average C_T_ t (ACTB)** | **∆ C_T_ = Target Gene - ACTB** | **∆∆ C_T_ = ∆ C_T_ Target Gene - ∆Ct Control** | **2^-∆∆^ ^CT^** |
| --- | --- | --- | --- | --- | --- | --- |
| **Parental H103** | **Oct4** | 33.53 ± 2.05 | 18.50 ± 0.79 | 15.03 ± 2.20 | 0 ± 2.20 | 1 |
|  | **Sox2** | 33.50 ± 1.12 | 18.50 ± 0.79 | 15.00 ± 1.37 | 0 ± 1.37 | 1 |
|  | **Klf4** | 27.87 ± 0.20 | 18.50 ± 0.79 | 9.37 ± 0.81 | 0 ± 0.81 | 1 |
|  | **C-Myc** | 25.13 ± 0.84 | 18.50 ± 0.79 | 6.63 ± 1.15 | 0 ± 1.15 | 1 |
|  | **Nanog** | 36.20 ± 0.29 | 18.50 ± 0.79 | 17.70 ± 0.84 | 0 ± 0.84 | 1 |
| **IPSC**  **H103**  **(P5)** | **Oct4** | 34.94 ± 1.64 | 18.79 ± 0.77 | 16.15 ± 1.81 | 1.12 ± 1.81 | 0.46 |
|  | **Sox2** | 27.97 ± 0.41 | 18.79 ± 0.77 | 9.18 ± 0.87 | -5.82 ± 0.87 | 56.49 |
|  | **Klf4** | 30.58 ± 2.12 | 18.79 ± 0.77 | 9.80 ± 2.25 | 2.42 ± 2.25 | 0.19 |
|  | **C-Myc** | 31.79 ± 0.25 | 18.79 ± 0.77 | 11.79 ± 0.81 | 5.16 ± 0.81 | 0.03 |
|  | **Nanog** | 34.57 ± 1.22 | 18.79 ± 0.77 | 15.78 ± 1.44 | -1.92 ± 1.44 | 3.78 |
| **IPSC**  **H103 (P10)** | **Oct4** | 31.99 ± 1.50 | 17.69 ± 0.19 | 14.30 ± 1.51 | -0.73 ± 1.51 | 1.66 |
|  | **Sox2** | 26.77 ± 0.82 | 17.69 ± 0.19 | 9.08 ± 0.84 | -5.92 ± 0.84 | 60.55 |
|  | **Klf4** | 30.74 ± 0.66 | 17.69 ± 0.19 | 13.05 ± 0.69 | 3.68 ± 0.69 | 0.08 |
|  | **C-Myc** | 31.09 ± 0.50 | 17.69 ± 0.19 | 13.40 ± 0.53 | 6.77 ± 0.53 | 0.01 |
|  | **Nanog** | 32.16 ± 1.17 | 17.69 ± 0.19 | 14.47 ± 1.19 | -3.23 ± 1.19 | 9.38 |

**Fold Change of Pluripotent Genes Expression in Reprogrammed H103 Relative to Parental by ∆∆Ct Method**

Sample

**REPLICATE** 3

|  | **Gene** | **Average C_T_ (target gene)** | **Average C_T_ t (ACTB)** | **∆ C_T_ = Target Gene - ACTB** | **∆∆ C_T_ = ∆ C_T_ Target Gene - ∆Ct Control** | **2^-∆∆^ ^CT^** |
| --- | --- | --- | --- | --- | --- | --- |
| **Parental H103** | **Oct4** | 33.19 ± 1.84 | 18.98 ± 0.65 | 14.21 ± 1.95 | 0 ± 1.95 | 1 |
|  | **Sox2** | 33.03 ± 1.15 | 18.98 ± 0.65 | 14.05 ± 1.32 | 0 ± 1.32 | 1 |
|  | **Klf4** | 27.97 ± 1.40 | 18.98 ± 0.65 | 8.99 ± 1.54 | 0 ± 1.54 | 1 |
|  | **C-Myc** | 24.77 ± 0.19 | 18.98 ± 0.65 | 5.79 ± 0.68 | 0 ± 0.68 | 1 |
|  | **Nanog** | 35.99 ± 1.23 | 18.98 ± 0.65 | 17.01 ± 1.40 | 0 ± 1.40 | 1 |
| **IPSC**  **H103**  **(P5)** | **Oct4** | 34.59 ± 0.35 | 18.89 ± 0.19 | 15.70 ± 0.40 | 1.49 ± 0.40 | 0.36 |
|  | **Sox2** | 27.31 ± 0.36 | 18.89 ± 0.19 | 8.42 ± 0.41 | -5.63 ± 0.41 | 49.52 |
|  | **Klf4** | 30.73 ± 1.10 | 18.89 ± 0.19 | 11.84 ± 1.12 | 2.85 ± 1.12 | 0.14 |
|  | **C-Myc** | 31.61 ± 0.40 | 18.89 ± 0.19 | 12.72 ± 0.44 | 6.93 ± 0.44 | 0.01 |
|  | **Nanog** | 35.02 ± 0.37 | 18.89 ± 0.19 | 16.13 ± 0.42 | -0.88 ± 0.42 | 1.84 |
| **IPSC**  **H103 (P10)** | **Oct4** | 31.95 ± 0.32 | 19.85 ± 0.25 | 12.1 ± 0.41 | -2.11± 0.41 | 4.32 |
|  | **Sox2** | 27.54 ± 1.40 | 19.85 ± 0.25 | 7.69 ± 1.42 | --6.36 ± 1.42 | 82.14 |
|  | **Klf4** | 31.07 ± 1.39 | 19.85 ± 0.25 | 11.22 ± 1.41 | 2.23 ± 1.41 | 0.21 |
|  | **C-Myc** | 30.27 ± 0.78 | 19.85 ± 0.25 | 10.42 ± 0.82 | 4.63 ± 0.82 | 0.04 |
|  | **Nanog** | 34.68 ± 0.59 | 19.85 ± 0.25 | 14.83 ± 0.64 | -.2.18 ± 0.64 | 4.53 |

**Fold Change of Pluripotent Genes Expression in Reprogrammed H103 Relative to Parental by ∆∆Ct Method**

Sample
