# Supplementary material for: Susceptibility of Human Oral Squamous Cell Carcinoma (OSCC) H103 and H376 cell lines to Retroviral OSKM mediated reprogramming
Source: PeerJ. 2017 Apr 13;5:e3174. doi: 10.7717/peerj.3174 (PMC5392249; doi:10.7717/peerj.3174)
Supplement: Data S2 — Quantitative data obtained by RT-qPCR from H376 RNA [file peerj-05-3174-s002.doc]

**REPLICATE 1**

|  | **Gene** | **Average C_T_ (target gene)** | **Average C_T_ t (ACTB)** | **∆ C_T_ = Target Gene - ACTB** | **∆∆ C_T_ = ∆ C_T_ Target Gene - ∆Ct Control** | **2^-∆∆^ ^CT^** |
| --- | --- | --- | --- | --- | --- | --- |
| **Parental H376** | **Oct4** | 30.21 ± 0.28 | 19.56 ± 0.63 | 10.65 ± 0.69 | 0 ± 0.69 | 1 |
|  | **Sox2** | 33.45 ± 1.12 | 19.56 ± 0.63 | 13.89 ± 1.29 | 0 ± 1.29 | 1 |
|  | **Klf4** | 27.94 ± 1.94 | 19.56 ± 0.63 | 8.38 ± 2.04 | 0 ± 2.04 | 1 |
|  | **C-Myc** | 27.56 ± 1.15 | 19.56 ± 0.63 | 8.00 ± 1.63 | 0 ± 1.63 | 1 |
|  | **Nanog** | 35.97 ± 0.65 | 19.56 ± 0.63 | 16.41 ± 0.91 | 0 ± 0.91 | 1 |
| **IPSC**  **H376**  **(P5)** | **Oct4** | 35.19 ± 0.66 | 21.93 ± 0.83 | 13.26 ± 1.06 | 2.61 ± 1.06 | 0.16 |
|  | **Sox2** | 32.36 ± 0.42 | 21.93 ± 0.83 | 10.43 ± 0.93 | -3.46 0.93 | 11.00 |
|  | **Klf4** | 32.40 ± 1.05 | 21.93 ± 0.83 | 10.47 ± 1.34 | 2.09 ± 1.34 | 0.23 |
|  | **C-Myc** | 33.50 ± 1.22 | 21.93 ± 0.83 | 11.57 ± 1.48 | 3.57 ± 1.48 | 0.08 |
|  | **Nanog** | 38.55 ± 0.45 | 21.93 ± 0.83 | 16.62 ± 0.94 | 0.21 ± 0.94 | 0.86 |
| **IPSC**  **H376 (P10)** | **Oct4** | 29.10 ± 0.36 | 17.17 ± 0.36 | 13.93 ± 0.51 | 1.28 ± 0.51 | 0.41 |
|  | **Sox2** | 25.11 ± 0.50 | 17.17 ± 0.36 | 7.94 ± 0.62 | -5.95 ± 0.62 | 61.82 |
|  | **Klf4** | 26.57 ± 0.93 | 17.17 ± 0.36 | 9.40 ± 1.0 | 1.02 ± 1.0 | 0.49 |
|  | **C-Myc** | 33.12 ± 2.11 | 17.17 ± 0.36 | 15.95 ± 2.14 | 7.95 2.14 | 0.00 |
|  | **Nanog** | 33.28 ± 0.45 | 17.17 ± 0.36 | 16.11 ± 0.58 | -0.30 ± 0.58 | 1.23 |

**Fold Change of Pluripotent Genes Expression in Reprogrammed H376 Relative to Parental by ∆∆Ct Method**

Sample

**REPLICATE** 2

|  | **Gene** | **Average C_T_ (target gene)** | **Average C_T_ t (ACTB)** | **∆ C_T_ = Target Gene - ACTB** | **∆∆ C_T_ = ∆ C_T_ Target Gene - ∆Ct Control** | **2^-∆∆^ ^CT^** |
| --- | --- | --- | --- | --- | --- | --- |
| **Parental H376** | **Oct4** | 30.14 ± 2.66 | 19.01 ± 0.10 | 11.13 ± 2.66 | 0 ± 2.66 | 1 |
|  | **Sox2** | 33.90 ± 1.70 | 19.01 ± 0.10 | 14.89 ± 1.70 | 0 ± 1.70 | 1 |
|  | **Klf4** | 28.03 ± 0.08 | 19.01 ± 0.10 | 9.02 ± 0.13 | 0 ± 0.13 | 1 |
|  | **C-Myc** | 27.34 ± 0.48 | 19.01 ± 0.10 | 8.33 ± 0.49 | 0 ± 0.49 | 1 |
|  | **Nanog** | 34.31 ± 0.29 | 19.01 ± 0.10 | 15.30 ± 0.31 | 0 | 1 |
| **IPSC**  **H376**  **(P5)** | **Oct4** | 34.82 ± 0.51 | 22.07 ± 0.86 | 12.75 ± 0.10 | 1.62± 0.10 | 0.33 |
|  | **Sox2** | 33.67 ± 0.23 | 22.07 ± 0.86 | 11.60 ± 0.89 | -3.29 ± 0.89 | 9.78 |
|  | **Klf4** | 32.31 ± 1.43 | 22.07 ± 0.86 | 10.24 ± 1.67 | 1.22 ± 1.67 | 0.43 |
|  | **C-Myc** | 35.80 ± 1.54 | 22.07 ± 0.86 | 13.73 ± 1.76 | 5.44 ± 1.76 | 0.03 |
|  | **Nanog** | 38.88 ± 0.24 | 22.07 ± 0.86 | 16.81 ± 0.89 | 1.51 ± 0.89 | 0.35 |
| **IPSC**  **H376 (P10)** | **Oct4** | 28.34 ± 0.61 | 17.00 ± 0.22 | 11.34 ± 0.65 | 0.21 ± 0.65 | 0.86 |
|  | **Sox2** | 26.12 ± 0.92 | 17.00 ± 0.22 | 9.12 ± 0.89 | -5.77 ± 0.89 | 54.57 |
|  | **Klf4** | 26.44 ± 0.69 | 17.00 ± 0.22 | 9.44 ± 0.72 | 0.42 ± 0.72 | 0.75 |
|  | **C-Myc** | 32.77 ± 1.38 | 17.00 ± 0.22 | 15.77 ± 1.40 | 7.44 ± 1.40 | 0.01 |
|  | **Nanog** | 31.21 ± 0.48 | 17.00 ± 0.22 | 14.21 ± 0.28 | -1.09 ± 0.28 | 2.13 |

**Fold Change of Pluripotent Genes Expression in Reprogrammed H376 Relative to Parental by ∆∆Ct Method**

Sample

**REPLICATE** 3

|  | **Gene** | **Average C_T_ (target gene)** | **Average C_T_ t (ACTB)** | **∆ C_T_ = Target Gene - ACTB** | **∆∆ C_T_ = ∆ C_T_ Target Gene - ∆Ct Control** | **2^-∆∆^ ^CT^** |
| --- | --- | --- | --- | --- | --- | --- |
| **Parental H376** | **Oct4** | 30.21 ± 0.45 | 19.78 ± 0.38 | 10.43 ± 0.59 | 0 ± 0.59 | 1 |
|  | **Sox2** | 33.69 ± 0.56 | 19.78 ± 0.38 | 13.91 ± 0.68 | 0 ± 0.68 | 1 |
|  | **Klf4** | 27.92 ± 2.07 | 19.78 ± 0.38 | 8.14 ± 2.10 | 0 ± 2.10 | 1 |
|  | **C-Myc** | 27.24 ± 0.40 | 19.78 ± 0.38 | 7.46 ± 0.30 | 0 ± 0.30 | 1 |
|  | **Nanog** | 34.11 ± 0.63 | 19.78 ± 0.38 | 14.33 ± 0.74 | 0 ± 0.74 | 1 |
| **IPSC**  **H376**  **(P5)** | **Oct4** | 35.57 ± 0.70 | 22.72 ± 0.19 | 12.85 ± 0.73 | 2.42 ± 0.73 | 0.19 |
|  | **Sox2** | 32.51 ± 0.84 | 22.72 ± 0.19 | 9.79 ± 0.86 | -4.12 ± 0.86 | 17.39 |
|  | **Klf4** | 32.58 ± 1.43 | 22.72 ± 0.19 | 9.86 ± 1.44 | 1.72 ± 1.44 | 0.30 |
|  | **C-Myc** | 33.33 ± 0.71 | 22.72 ± 0.19 | 10.61 ± 0.73 | 3.15 ± 0.73 | 0.11 |
|  | **Nanog** | 38.11 ± 0.50 | 22.72 ± 0.19 | 15.39 ± 0.53 | 1.06 ± 0.53 | 0.48 |
| **IPSC**  **H376 (P10)** | **Oct4** | 30.21 ± 0.42 | 18.82 ± 0.09 | 11.39 ± 0.43 | 0.96 ± 0.43 | 1.95 |
|  | **Sox2** | 26.97 ± 0.85 | 18.82 ± 0.09 | 8.15 ± 0.85 | -5.76 ± 0.85 | 54.19 |
|  | **Klf4** | 28.45 ± 0.58 | 18.82 ± 0.09 | 9.63 ± 0.59 | 1.49 ± 0.59 | 0.36 |
|  | **C-Myc** | 33.47 ± 0.50 | 18.82 ± 0.09 | 14.65 ± 0.51 | 7.19 ± 0.51 | 0.01 |
|  | **Nanog** | 31.99 ± 2.60 | 18.82 ± 0.09 | 13.17 ± 2.60 | -1.16 ± 2.60 | 2.23 |

**Fold Change of Pluripotent Genes Expression in Reprogrammed H376 Relative to Parental by ∆∆Ct Method**

Sample
