# Supplementary material for: Susceptibility of Human Oral Squamous Cell Carcinoma (OSCC) H103 and H376 cell lines to Retroviral OSKM mediated reprogramming
Source: PeerJ. 2017 Apr 13;5:e3174. doi: 10.7717/peerj.3174 (PMC5392249; doi:10.7717/peerj.3174)
Supplement: Supplemental Information 1 — Post-transfection of 293FT at 48 hours. (A–B) 293FT cell line transfected with retro—pMX GFP plasmid served as positive control. (C-D)Transfection of 293FT without retro-pMX GFP used as negative control. Zeiss Axiovert inverted microscope, original magnification: 10×. (PPT). Supplementary Figure 2. Transduction of (A–B) H103 and (C-D) H376 without retro-pMX GFP used as negative control. No GFP signals detected. Zeiss Axiovert inverted microscope, original magnification: 20x. (PPT) . Supplementary Table 1. [file peerj-05-3174-s003.pdf]

Supplementary Figure 1

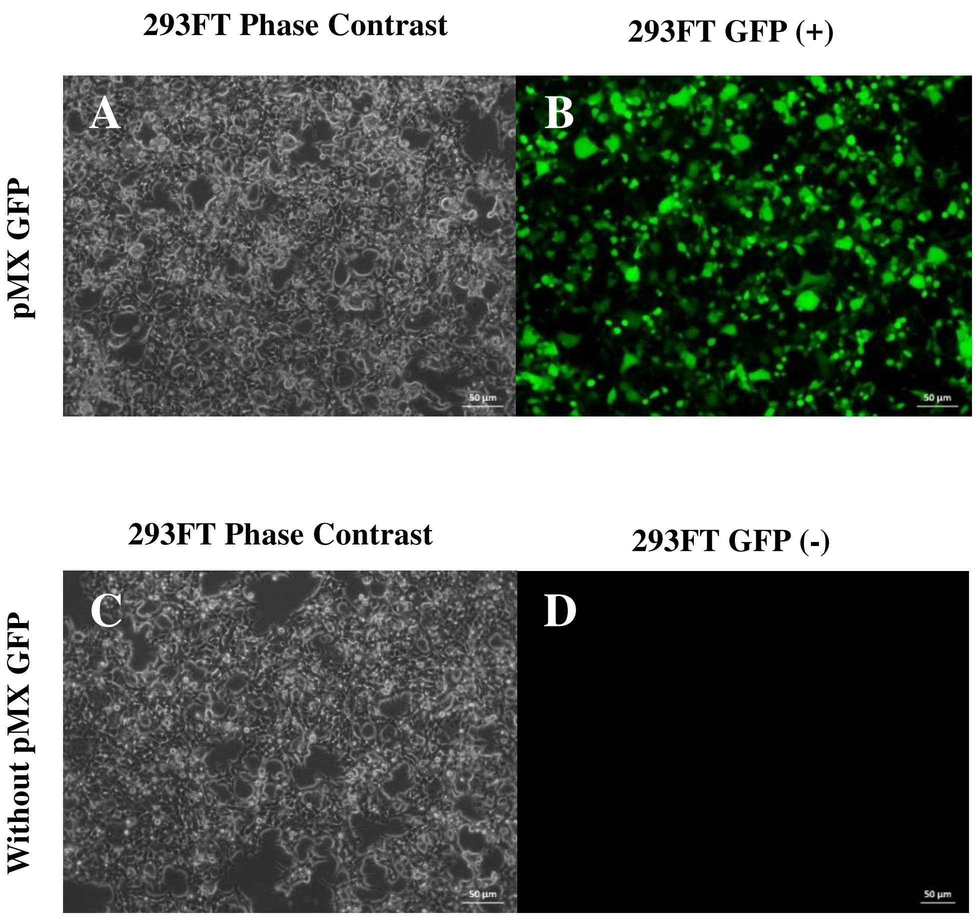

S1 Figure. Post-transfection of 293FT at 48 hours. (A-B) 293FT cell line transfected with retro - pMX GFP plasmid served as positive control. (C-D)Transfection of 293FT without retro-pMX GFP used as negative control. Zeiss Axiovert inverted microscope, original magnification: 10x. (PPT)

Supplementary Figure 2

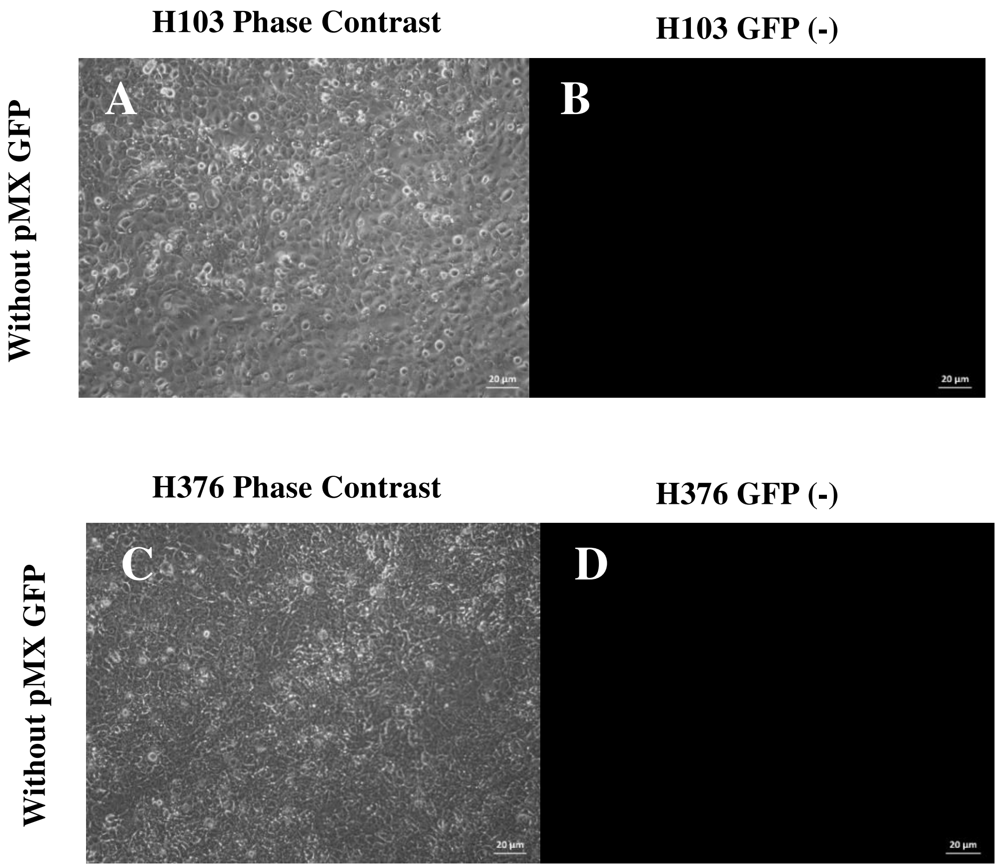

S2 Figure. Transduction of (A-B) H103 and (C-D) H376 without retro-pMX GFP used as negative control. No GFP signals detected. Zeiss Axiovert inverted microscope, original magnification: 20x. (PPT)

Supplementary Table 1

NO	Gene Symbol	Forward Primer	Reverse Primer
1	GAPDH	AGA GGC AGG GAT GAT GTT CT	TCT GCT GAT GCC CCC ATG TT
2	OCT4	GAC AGG GGG AGG GGA GGA GCT AGG	CTT CCC TCC AAC CAG TTG CCC CAA AC
3	SOX 2	GGG AAA TGG GAG GGG TGC AAA AGA GG	TTG CGT GAG TGT GGA TGG GAT TGG TG
4	KLF4	ACG ATC GTG GCC CCG GAA AAG GAC C	TGA TTG TAG TGC TTT CTG GCT GGG CTC C
5	C-MYC	GCG TCC TGG GAA GGG AGA TCC GGA GC	TTG AGG GGC ATC GTC GCG GGA GGC TG
6	NANOG	TTT GGA AGC TGC TGG GGA AG	GAT GGG AGG AGG GGA GAG GA
